# Microprolactinoma Growth During Pregnancy With Pituitary Tumor Apoplexy: Case Report and Review of the Literature

**DOI:** 10.1155/crie/2490132

**Published:** 2025-01-07

**Authors:** Lucia Introini, Jenifer Silva, Mariana Risso, Beatriz Mendoza, Maria M. Pineyro

**Affiliations:** Unidad Académica de Endocrinología y Metabolismo, Hospital de Clínicas “Dr. Manuel Quíntela”, Facultad de Medicina, Universidad de la República, Montevideo, Uruguay

## Abstract

Prolactinomas are the most prevalent subtype of pituitary adenomas and represent one of the leading etiological factors responsible for amenorrhea and infertility in women. The primary therapeutic approach entails the use of dopamine agonists, which effectively restore fertility. In cases of microprolactinomas, the likelihood of experiencing a symptomatic enlargement of the tumor during pregnancy is exceptionally low, estimated at a mere 2.4%. Consequently, once pregnancy is successfully achieved, the administration of dopamine agonists is discontinued, with ongoing clinical monitoring of the patient's condition. The incidence of pituitary apoplexy during pregnancy is exceedingly rare. We present a case of a 29-year-old patient with microprolactinoma, treated with cabergoline, which was discontinued upon achieving pregnancy. However, at the 16th week of gestation, she presented with persistent headaches and compromised visual acuity, manifesting as left temporal hemianopia. A noncontrast magnetic resonance imaging (MRI) revealed an enlargement of the adenoma, accompanied by evidence of hemorrhage. She was referred to our hospital at 26 weeks of gestation. Cabergoline treatment was reinstated and well tolerated by the patient. Doses were increased to 2 mg per week because bitemporal hemianopia was not improving. A subsequent noncontrast MRI scan performed at 35 weeks of gestation demonstrated a further increase in adenoma size, measuring 17 × 21 × 13 mm, with signs of intratumoral bleeding. A planned cesarean section was performed at 39 weeks of gestation, without encountering maternal–fetal complications. Breastfeeding was not initiated due to the adenoma's compression of the chiasm. Accordingly, dopamine agonist therapy was continued. During the postpartum follow-up, the patient experienced a resumption of menstrual cycles, normalization of prolactin levels, and a reduction in tumor size. Ultimately, the diagnosis was established as a microprolactinoma, which had enlarged during pregnancy due to a pituitary tumor apoplexy. Although microprolactinomas typically carry a low risk of symptomatic tumor growth during pregnancy, this case emphasizes the critical importance of vigilant clinical monitoring to swiftly detect and manage this rare complication. This instance serves as an educational example of an uncommon event—a microprolactinoma experiencing apoplexy during pregnancy.

## 1. Introduction

Prolactinomas are tumors derived from pituitary lactotroph cells and represent the most common pituitary adenomas, accounting for 40%–50% of cases. They have a higher incidence in women aged between 25 and 35, with the majority being microprolactinomas (less than 10 mm) [1, 2]. These tumors often manifest as infertility since hyperprolactinemia affects the secretion of gonadotropin-releasing hormone (GnRH), follicle-stimulating hormone (FSH), and luteinizing hormone (LH). Moreover, elevated prolactin levels hinder corpus luteum formation and the secretion of progesterone. All this leads to amenorrhea, infertility, and hypogonadism [2–5]. Effective treatment with dopamine agonists such as bromocriptine and cabergoline can reverse hyperprolactinemia and hypogonadism while promoting a reduction in tumor size in most cases, thus facilitating pregnancy [4–8]. In microprolactinomas, the risk of symptomatic tumor enlargement during pregnancy is very low (2.4%), with minimal growth in the maximum adenoma diameter, typically not exceeding 10 mm. After achieving pregnancy, dopamine agonists are discontinued, and the patient is closely monitored clinically. Pituitary apoplexy during pregnancy is exceedingly rare, with a reported prevalence of 1 in 10,000 pregnancies [9–11]. It presents as an acute clinical syndrome resulting from tumor infarction or hemorrhage, causing a sudden and transient increase in size. Here, we present the case of a patient with a microprolactinoma who experienced significant growth during pregnancy due to intratumoral bleeding (apoplexy).

## 2. Case Presentation

**Figure 1:**
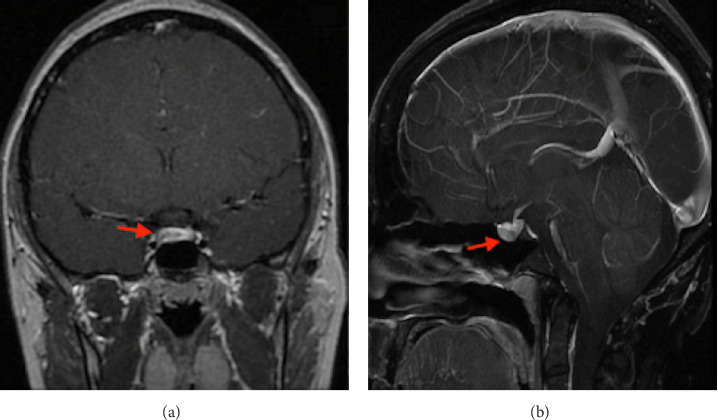
Magnetic resonance imaging, T1 post-gadolinium. (A) Coronal view and (B) Sagittal view. A 3 × 4.6 × 3.7 mm microadenoma is observed (red arrow).

A 29-year-old woman from Venezuela had a medical history of hyperprolactinemia diagnosed in her country of origin, with no prior imaging or treatment. Since relocating to Uruguay 5 years ago, she has not undergone any follow-up care. She presented herself at another hospital with a 3-year history of amenorrhea and a strong desire for pregnancy. She denied experiencing galactorrhea or headaches and was not taking any medications at the time of consultation. Her prolactin levels were elevated at 157.4 ng/ml (normal reference range: 4.8–23.3 ng/ml). Her liver and renal function, as well as TSH levels, were within normal limits. A pituitary magnetic resonance imaging (MRI) scan revealed a hypointense area on T1-weighted images following gadolinium administration, measuring 3 × 4.6 × 3.7 mm, consistent with a microadenoma. The pituitary stalk showed a slight deviation to the left (Figure 1).

**Figure 2:**
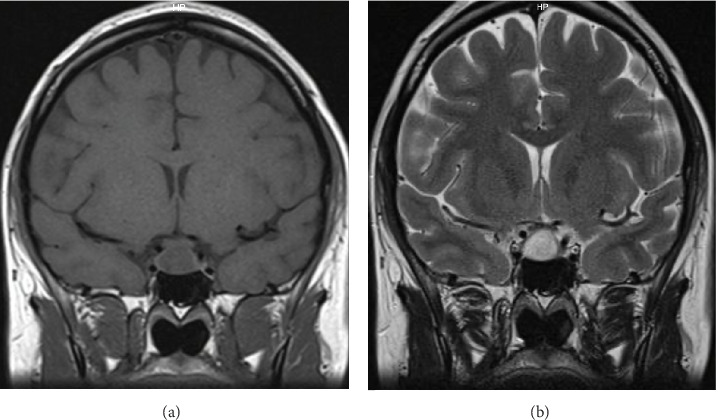
MRI, (A) T1 coronal view without contrast and (B) T2. An increase in tumor size is observed, measuring 12 × 19 × 11 mm. with intratumoral bleeding.

Initial treatment with 5 mg of bromocriptine was initiated; however, her prolactin levels continued to rise, reaching 212 ng/ml. Subsequently, she was transitioned to cabergoline in incremental doses, ultimately reaching 1 mg per week. Pregnancy was successfully achieved with prolactin levels decreasing to 49 ng/ml, leading to the discontinuation of cabergoline. At 17 weeks of gestation, she presented with persistent headaches and altered visual acuity. A confrontational visual field examination revealed left temporal hemianopsia. A pituitary MRI without contrast demonstrated an enlargement of the pituitary adenoma, measuring 12 × 19 × 11 mm, with extension into the suprasellar cistern and compression of the optic chiasm. The pituitary stalk was no longer visible, and there was no evidence of cavernous sinus invasion; however, intratumoral bleeding was observed (Figure 2). The patient was referred to our hospital.

Her initial evaluation at our facility took place at 26 weeks of gestational age. Cortisol and thyroid hormone levels were within normal limits. Treatment with cabergoline was reinstated, with the dosage progressively increased to 2 mg per week. Ophthalmological evaluation confirmed bilateral temporal hemianopsia.

**Figure 3:**
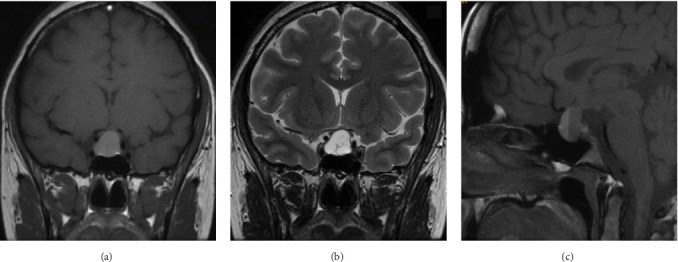
MRI without contrast. (A) T1 coronal view, (B) T2 and (C) T1 sagittal view. An increase in the size of the prolactinoma is observed, measuring 17 × 21 × 13 mm, compressing the chiasm. Presents fluid level and is hyperintense on T1 compatible with bleeding.

Given the persistence of headaches and the absence of significant improvement in visual acuity, a follow-up MRI scan, conducted without contrast, was scheduled at 35 weeks of gestation. This scan revealed further enlargement of the adenoma to 17 × 21 × 13 mm, accompanied by hemorrhage within the pituitary lesion (Figure 3).

Thyroid function tests and cortisol levels remained within the normal range.

**Figure 4:**
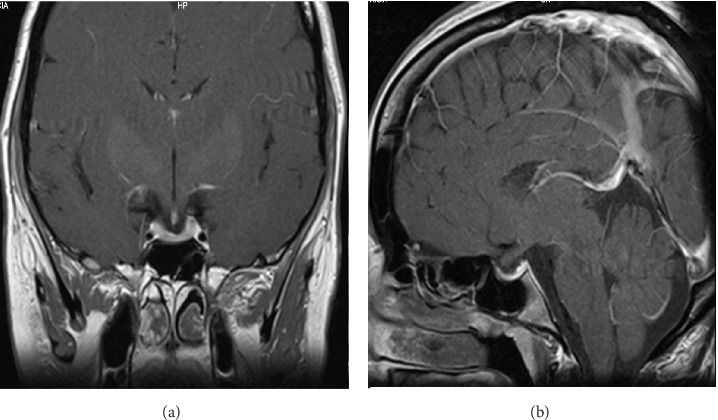
Magnetic resonance imaging, T1 post-gadolinium. (A) Coronal view and (B) Sagittal view. No evidence of the prolactinoma is seen.

In collaboration with the obstetrics team, it was decided to proceed with a scheduled cesarean section upon completion of 39 weeks of gestation. The male newborn had a birth weight of 3686 g, a length of 51 cm, and a head circumference of 36.5 cm, all appropriate for gestational age. No perinatal complications were observed, and they were discharged 72 h after birth. Due to the chiasm compression, breastfeeding was not initiated, and dopamine agonist therapy was continued. Subsequently, the patient resumed regular menstrual cycles, prolactin levels normalized, and there was no evidence of tumor remnant on MRI (Figure 4).

## 3. Discussion

The occurrence of hemorrhage or infarction within a pituitary tumor, known as pituitary apoplexy, represents a rare and potentially life-threatening complication, often carrying serious consequences, including deficiencies in hormonal axes originating from the pituitary gland [2]. The estimated prevalence of this condition is ~6.2 cases per 100,000 individuals in the general population, with a slightly higher occurrence of 1 per 10,000 in pregnant women [9–11]. Within the subgroup of prolactinomas, the prevalence of pituitary apoplexy is roughly 6.8%, with a higher incidence observed in macroprolactinomas (20.3%) compared to microprolactinomas (3.1%) [12].

Pregnancy represents a recognized risk factor for the development of pituitary apoplexy. This heightened risk can be attributed to increased blood flow and hormonal stimulation of the pituitary gland, resulting in an enlargement of both the pituitary gland itself and any preexisting tumors [13]. Furthermore, during pregnancy, the blood supply to the pituitary gland may be compromised due to the expansion of lactotroph cells and the consequent compression of blood vessels [14]. It is a matter of debate whether treatment with dopamine agonists can induce pituitary apoplexy [15]. Other predisposing factors encompass arterial hypertension, diabetes, discontinuation of dopamine agonist therapy, head trauma, use of estrogen therapy, and anticoagulant medications [9].

There have been 48 documented cases of pituitary apoplexy occurring during pregnancy, with only nine of these cases involving patients with microprolactinomas, as in our case (Table 1) [9, 16, 17].

Notably, it has been reported that pituitary apoplexy tends to occur more frequently during the second and third trimesters of pregnancy [9, 16]. In all instances involving microprolactinomas, dopamine agonists were discontinued upon pregnancy confirmation. The most common initial symptoms were sudden severe headaches, often followed by visual disturbances (present in seven cases of microprolactinomas, including our case).

In cases of severe and intractable headaches, it is advisable to perform an MRI without contrast and hormonal studies to assess for potential pituitary hormone deficiencies. In our patient's case, despite the detection of pituitary hemorrhage, other pituitary hormonal axes remained unaffected, as indicated by normal cortisol and free thyroxin levels, obviating the need for exogenous supplementation.

In situations where neurological status deteriorates, surgical decompression may become necessary, typically during the second trimester [2, 5]. Presently, there are no established clinical guidelines for the management of pituitary apoplexy during pregnancy. Optimal management should be guided by a multidisciplinary team, including an endocrinologist, ophthalmologist, and neurosurgeon, and tailored to the patient's individual characteristics [13]. The primary objective is to alleviate symptoms and relieve compression of the optic pathway.

In the United Kingdom, guidelines for managing pituitary apoplexy recommend surgical intervention in cases exhibiting significant ophthalmic signs or a decreased level of consciousness. Additionally, they propose the initiation of steroid therapy empirically for hemodynamically unstable patients and consider steroid therapy for stable patients with morning cortisol levels below 19 mcg/dl [18]. Surgical procedures during pregnancy have been documented in 12 cases (48%), while the remaining cases, including ours, were managed conservatively. Conservative management typically includes the use of dopamine agonists in eight cases and hormone replacement therapy when deemed necessary. Importantly, most reported cases, including ours, culminated in full-term pregnancies, with all newborns exhibiting good health [9, 16].

Regarding the management of microprolactinomas during pregnancy, current guidelines recommend discontinuing treatment with dopamine agonists upon confirming pregnancy. This is due to the fact that these drugs can cross the placental barrier, potentially exposing the developing fetus to their effects [1]. Among the available dopamine agonists, bromocriptine has undergone extensive safety studies and has demonstrated a favorable safety profile. Studies have reported rates of spontaneous abortions, fetal malformations, and alterations in postnatal development comparable to those observed in healthy women [2, 4]. Cabergoline, which boasts a longer half-life, also appears to be safe for use during pregnancy. However, there is a relative paucity of studies compared to bromocriptine regarding its safety in pregnancy.

A recent study conducted in Brazil, involving 223 pregnancies in patients with prolactinomas, reported a spontaneous abortion rate of 11%, a figure similar to that in the general population. Interestingly, the study revealed higher rates of spontaneous abortion in patients who continued cabergoline treatment after pregnancy detection compared to those who discontinued treatment (38% vs., 7.5%) [8]. Notably, there were no significant differences in terms of other maternal–fetal outcomes. Importantly, current evidence does not suggest harm to fetuses exposed to dopamine agonists [1, 2]. However, it is worth noting that more research is needed to comprehensively assess the safety of dopamine agonists during pregnancy.

Regarding the follow-up of prolactinomas during pregnancy, clinical monitoring of patients in each trimester is recommended. Measurement of prolactin levels during pregnancy is not advisable since physiologically, these levels can increase up to 10 times the normal range, with levels at the end of pregnancy reaching ~200 ng/ml [1, 2, 6]. This physiological increase is attributed to the proliferation of lactotroph cells and their hyperplasia, which leads to an enlargement of the pituitary gland, with dimensions reaching up to 11.8 mm at the end of pregnancy and immediately postpartum. This hormonal surge is mediated by placental estrogen stimulating the mitotic activity of lactotroph cells and the synthesis of prolactin [5, 6].

Routine MRI studies are also not recommended unless the patient presents with symptoms suggestive of adenoma growth, such as headaches or impaired visual acuity, as symptomatic growth of microprolactinomas is uncommon during pregnancy [1, 3]. The risk of a significant increase in tumor size is low, with complications in microprolactinomas, such as headaches or compression of the optic chiasm or pituitary stalk, occurring in less than 5% of cases [5, 6]. In contrast, macroprolactinomas carry a higher risk of symptomatic growth, with rates of up to 30% [4, 5].

In cases where clinical suspicion arises regarding tumor growth, an MRI without contrast should be performed. If tumor growth is confirmed, treatment with bromocriptine is typically recommended [1, 4]. In our specific case, cabergoline was reinstated as the patient had not responded satisfactorily to bromocriptine prior to pregnancy. Importantly, current literature does not indicate any additional harm to the fetus in such cases. If, following the restart of dopamine agonist treatment, the tumor size fails to decrease or symptoms due to mass effect persist, surgical intervention in the second trimester is a viable option, or delivery may be considered if the pregnancy is near term [1, 10].

Regarding breastfeeding, it is considered safe for patients who are not receiving dopamine agonists. However, it is not recommended for patients with prolactinomas that compress the optic chiasm or in cases where the use of dopamine agonists during pregnancy has been deemed necessary, as was the case in our situation [4, 5]. Following childbirth, prolactin levels decline rapidly, although they remain elevated during the lactation period. Importantly, breastfeeding has not been associated with an increase in tumor size, despite the fact that nipple stimulation can lead to increased prolactin secretion [5].

A recommended follow-up includes prolactin and MRI studies 2 months postpartum [4]. Interestingly, pregnancy has been shown to induce remission of hyperprolactinemia in microprolactinomas, likely due to autoinfarction of the tumor [19, 20]. In cases where remission is achieved, and dopamine agonist treatment is discontinued, regular monitoring is essential for at least 5 years [3]. Factors favoring remission include older maternal age, lower prolactin levels at diagnosis and postpartum, and smaller adenoma size at diagnosis [21].

## 4. Conclusion

While the likelihood of experiencing a symptomatic increase in tumor size in microprolactinomas during pregnancy is notably low, it remains imperative to maintain clinical follow-up to promptly detect and assess this potential complication. Furthermore, this case serves as a noteworthy illustration of a rare occurrence: a prolactinoma apoplexy during pregnancy.

## Figures and Tables

**Table 1:** Cases of pituitary apoplexy in microprolactinomas.

Reference	Age	Parity	Gestational age (weeks)	Adenoma size (mm)	PRL at diagnosis (ng/ml)	PRL before pregnancy (ng/ml)	Prior DA treatment	Clinical presentation	Therapeutic management	Birth	Lactation	Post partum treatment
Gondim et al. [22]	29	1	30	3 × 4	177	5	BC	Headache, decreased AV LE: central scotoma RE: Ptosis, diplopia, 3rd CN paralysis	BC for 2 weeks, then TSS, T4, HC	VD, term, healthy NB	No data	Did not require

Couture et al. [23]	37	1	16	7 × 7	105	35.4	CB	Headache, vomiting, blurred vision VF: normal	CB (up to 4 weeks after birth)	CS, term, healthy NB	Yes	Did not require

Hayes et al. [24]	41	1	18	6 × 9	61^a^	No data	CB	Headache, decreased VA VF: bitemporal deficit	TSS, HC	VD, term, healthy NB	Yes (2 weeks)	Did not require

Annamalai et al. [25]	25	1	37	4	96	No data	CB	Headache VF: normal	CB, HC	CS, term, healthy NB	Yes (CB suspended)	Did not require

Kuhn et al. [16]	31	2	36	No data	149	17	CB	Headache, photophobia VF: hemianopsia bitemporal	CB, TSS	CS, term, healthy NB	No data	No data

Kuhn et al. [16]	21	1	26	7	80	No data	BC	Fatigue, Polyurodipsia VF: normal	T4, HC, Desmopressin	VD, healthy NB	No data	TSS Desmopressin (DA)

Kuhn et al. [16]	32	3	33	10	240	No data	CB	Headache, decreased VA in LE	CB, HC	PV, term, healthy NB	No	CB, HC (SAI)

Kuhn et al. [16]	23	1	16	5	160	No data	CB	Headache, VF: quadrantopsia RE	CB	CS, term, healthy NB	Yes	TSS

Kuhn et al. [16]	25	2	24	No data	No data	No data	CB	Headache, VF: no data	CB	VD, healthy NB	No	TSS

Our case	29	1	16	3 × 4, 6 × 3.7	157.4	49	CB	Headache VF: temporal hemianopsia LE	CB	Cs, term, healthy	No	CB

Abbreviations: BC, bromocriptine; CB, cabergoline; CS, caesarean section; DA, dopamine agonist; HC, hydrocortisone; LE, left eye; NB, new born; RE, right eye; SAI, secondary adrenal insufficiency; T4, levothyroxine; TSS, transsphenoidal surgery; VA, visual acuity; VD, vaginal delivery; VF, visual field.

^a^1300 mUI/L.

## Data Availability

All relevant data underlying the findings of this case report are included within the article. No additional source data are available. The case information has been appropriately anonymized to protect patient confidentiality in accordance with ethical guidelines.
